# Supplementary material for: Structural basis for the rescue of hyperexcitable cells by the amyotrophic lateral sclerosis drug Riluzole
Source: Nat Commun. 2024 Sep 28;15:8426. doi: 10.1038/s41467-024-52539-4 (PMC11438954; doi:10.1038/s41467-024-52539-4)
Supplement: Supplementary file 1 — Supplementary Information [file 41467_2024_52539_MOESM1_ESM.pdf]

Supplementary Information for

Structural basis for the rescue of hyperexcitable cells by the Amyotrophic Lateral Sclerosis drug Riluzole

David Hollingworth, Frances Thomas, Dana A. Page, Mohamed A. Fouda, Raquel Lopez-Rios De Castro, Altin Sula, Vitaliy B. Mykhaylyk, Geoff Kelly, Martin B. Ulmschneider, Peter C. Ruben, B.A. Wallace

This File contains the Supplementary Figures 1-8 and Supplementary Tables 1-4

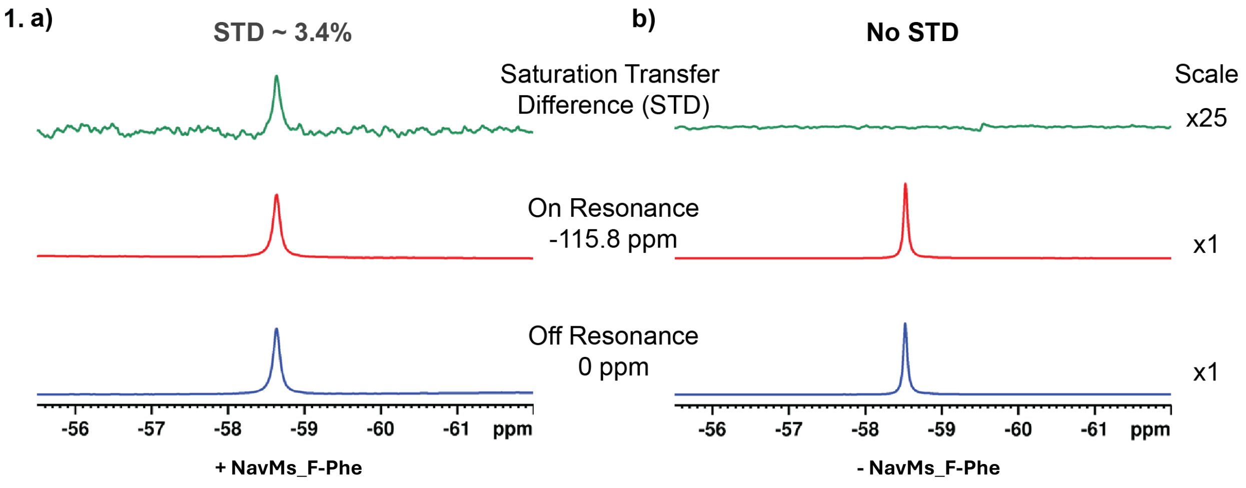

Supplementary Figure 1 Riluzole Interacts directly with NavMs

19F-19F saturation transfer occurs in the presence of NavMs_F-Phe but not in its absence. a), Irradiation at the fluorine resonance for NavMs_F-Phe (-119 ppm) produces STD at the riluzole fluorine resonance in the presence of NavMs_F-Phe b), no STD is produced in the same experiment without protein. Both experiments report STD at the riluzole 19F resonance after a 4-second saturation time.

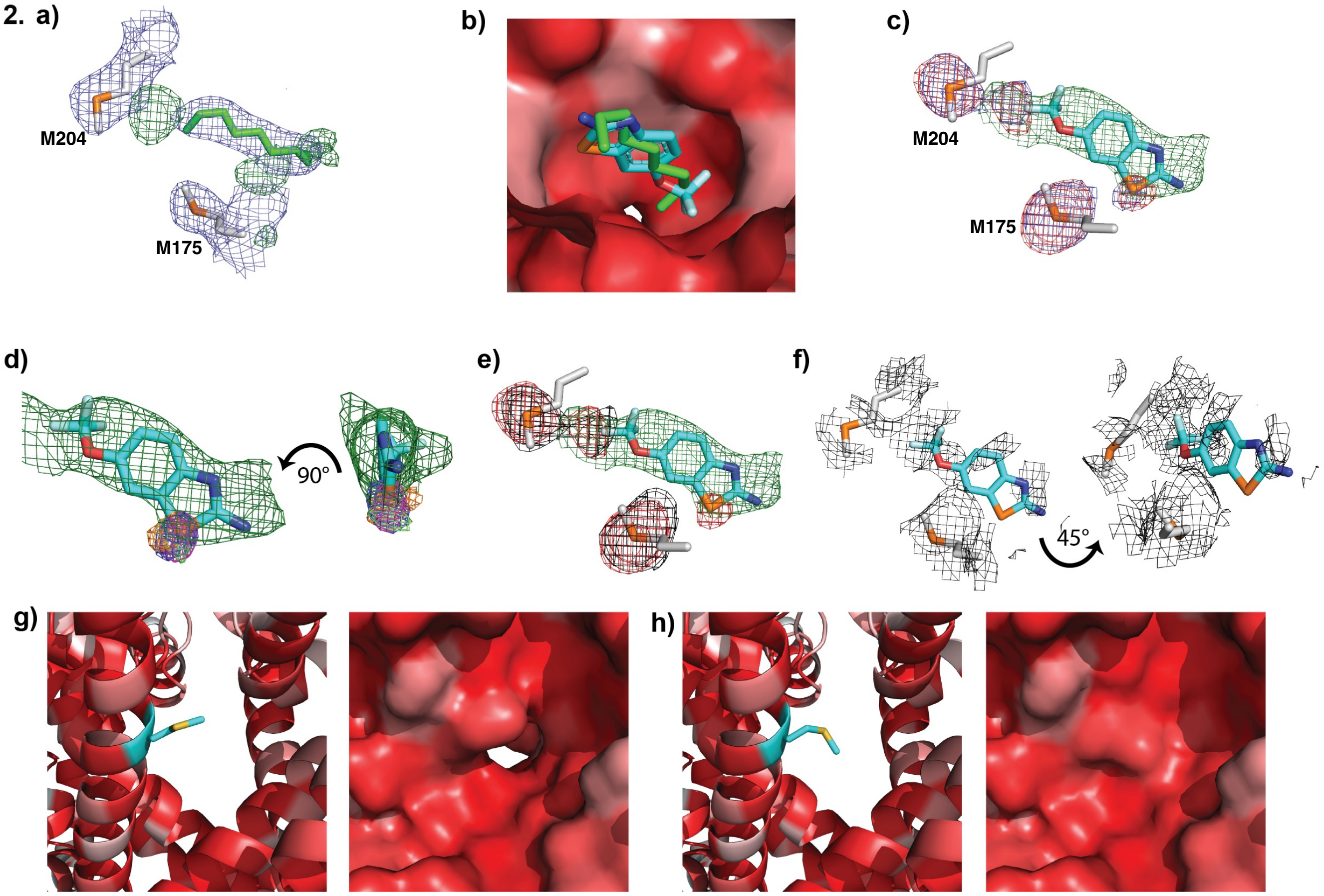

Supplementary Figure 2 X-ray crystallography identifies fenestration binding site of riluzole in NavMs

a) Fo-Fc difference density (green, 3σ) showing extra electron density around the fenestration ligands in riluzole co-crystals. 2Fo-Fc electron density map (blue, 1σ) b) placement of riluzole (coloured by heteroatom) into fenestration density results in almost perfect overlap with hega-10 (green stick) c) anomalous sulphur signals in fenestrations of co-crystals (contoured @3σ, red crystal 1, blue crystal 2) overlayed onto omit map (green) showing anomalous signals for Met204 (split signal), Met175, and riluzole d) anomalous signals for riluzole are maintained over 4 data collections on crystal 2 (1st blue @3σ, 2nd pink@3σ, 3rd orange @2.5σ, 4th green @2.5σ) e) DMSO only crystals do not contain the riluzole anomalous peak (red - co-crystal 1 @3σ, black - DMSO only crystal @3σ f) two views of the anomalous signal of DMSO-only crystals at noise level 1σ) (g and h) Sidechain movement closes fenestrations g) Met204 showing the open fenestration as the major form (h) sidechain movement closes the fenestration in the minor form.
Solid surface images of NavMs coloured by hydrophobicity scale from white to red as shown in main article Figure 2f.

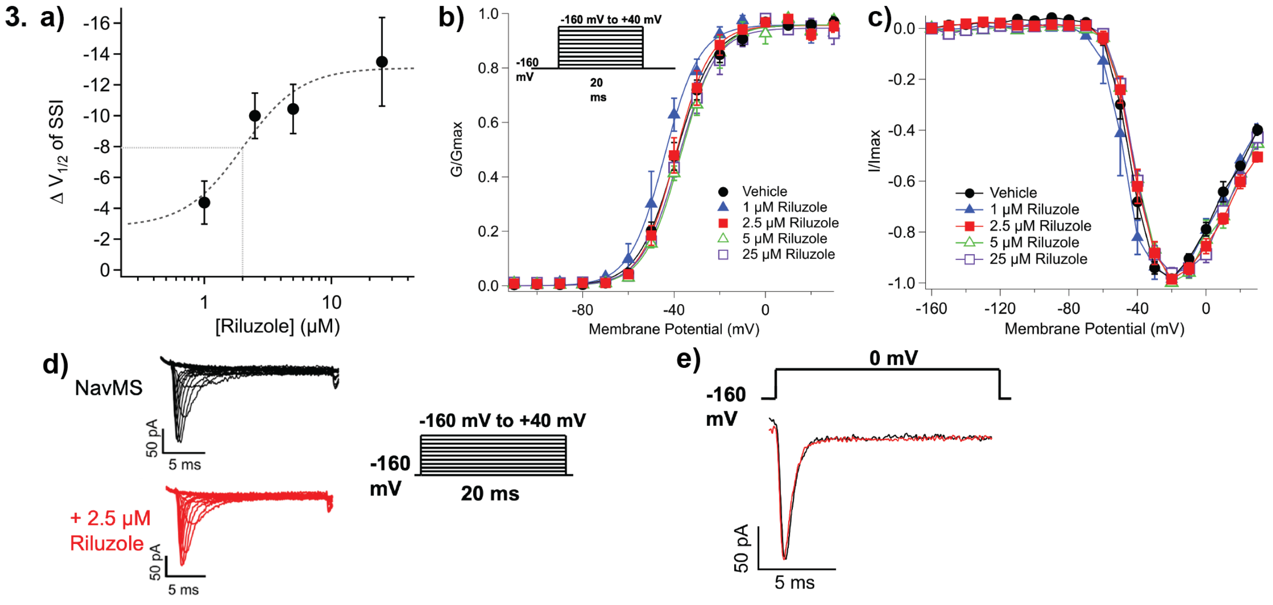

Supplementary Figure 3 – Riluzole selectively stabilises the inactivated state of NavMs

a) Shift in SSI $V_{1/2}$ for 1 – 25 μM riluzole were fit to the Hill equation, with a baseline fixed to $\Delta V_{1/2}$ of vehicle: -2.8 mV. The IC50 was 2.0 μM with a Hill coefficient of 1.9. b) Effect of riluzole or vehicle (black) on activation $V_{1/2}$ plotted as average ± S.E.M. with inset showing activation protocol used. Values were fit to a Boltzmann function. One-way Anova analysis found no significant difference in $V_{1/2}$ of activation between vehicle and any concentration of riluzole ($p > 0.5$) c) IV curves plotted as average ± S.E.M. d) Representative traces before (black) and after application 2.5 μM riluzole (red), protocol shown on right e) Superimposition of 0 mV traces from (d) show no channel block with 2.5 μM riluzole.

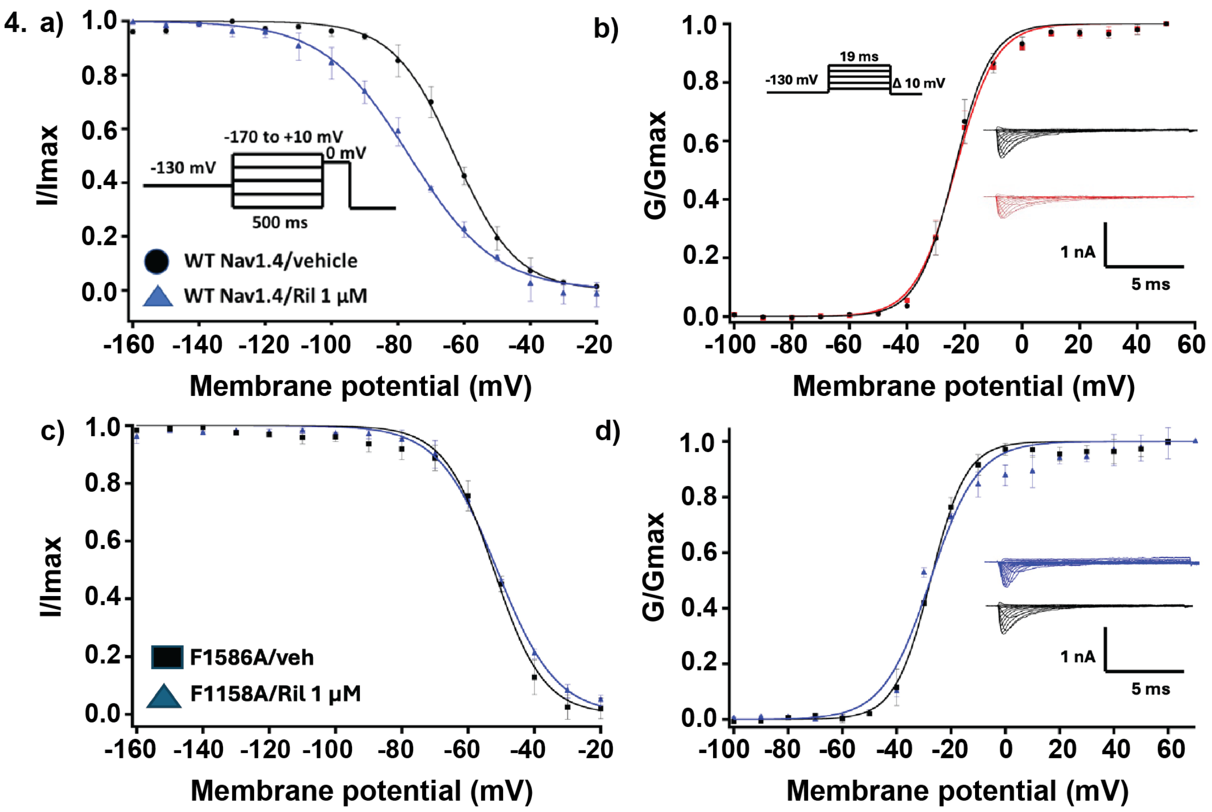

Supplementary Figure 4 Selective stabilisation of inactivation by riluzole on Nav1.4 is abolished by F1586A

a) Effect of 1 mM riluzole (blue triangle) or vehicle (black circle) on SSFI of WT Nav1.4 plotted as average (± S.E.M) with the insert showing the protocol (n = 5, each). Data points were fit to a Boltzmann function. b) Effect of 100 mM riluzole (blue triangle) or vehicle (black circle) on activation $V_{1/2}$ with inset showing protocol and representative currents (n = 5, each). Data points were fit to a Boltzmann function. c) Effect of 1 μM riluzole (blue triangle) or its vehicle (black circle) on SSFI of Nav1.4 F1586A plotted as average (± S.E.M), protocol as inset in (a), (n=5, each). Data points were fit to a Boltzmann function. d) Effect of 1 μM riluzole (blue triangle) or vehicle (black square) on Nav1.4 F1586A on activation $V_{1/2}$ with inset showing representative currents, protocol as inset in (b), (n = 5, each)

5.

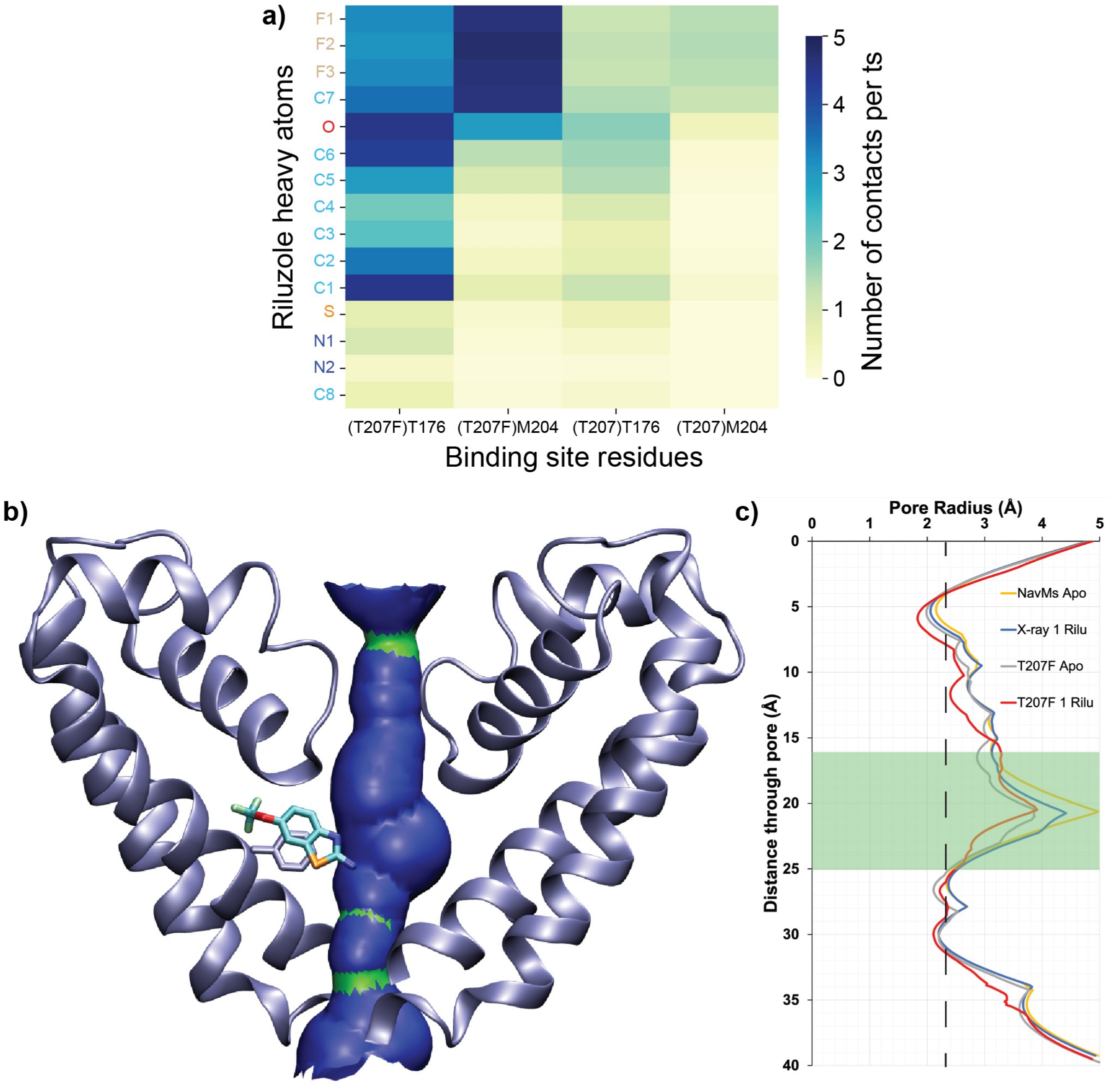

Supplementary Figure 5 The NavMs T207F mutant fenestration favours riluzole binding over wild-type fenestrations with binding maintaining the non-blocking pose

a) atomic contacts for riluzole binding in the eNav DIII-DIV mimicking fenestration of NavMs T207F) (left two panels) showing that contacts are much greater compared to those in the WT fenestrations of NavMs (right two panels) in the same simulation b) HOLE2 analysis showing that riluzole binding to NavMs T207F fenestration maintains the non-blocking profile of the WT channel. A pore radius of >2.3Å is required for Na+ conduction and is represented by blue in the channel tunnel cartoon (left panel) and delineated by the broken vertical line in the plot of pore radius along the pore axis (right panel). The green shaded area in the plot (right panel) represents the ion conduction pathway at fenestration depth.

6.

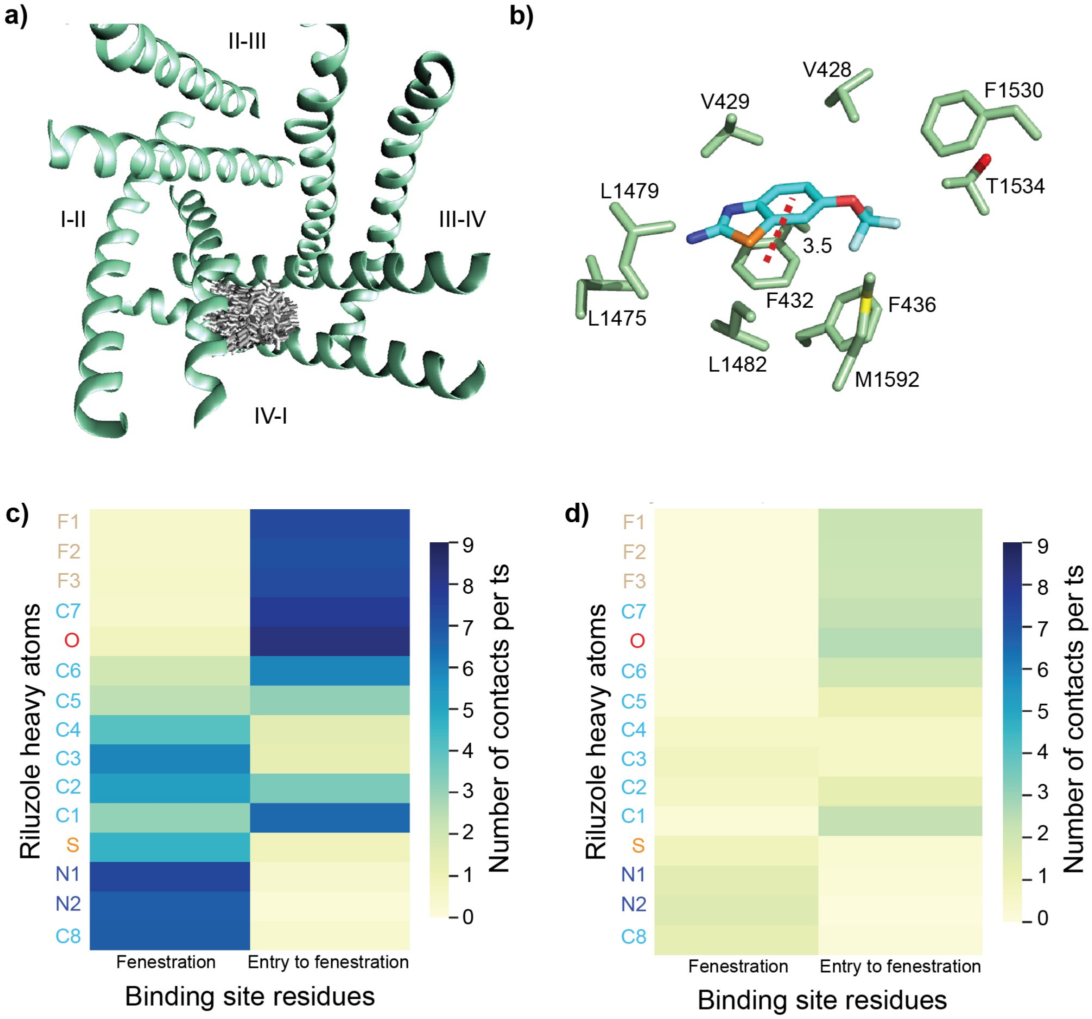

Supplementary Figure 6 – Riluzole binding in the fenestrations of hNav1.4 and hNav1.4 (F1586A)

a) MD simulation produces a binding cluster in the DIV-DI fenestration of hNav1.4. b) riluzole binding site for this cluster. c and d) contact maps for riluzole interaction in the DIII-DIV fenestration of (c) WT (d) and F1586A hNav1.4 channels

7.

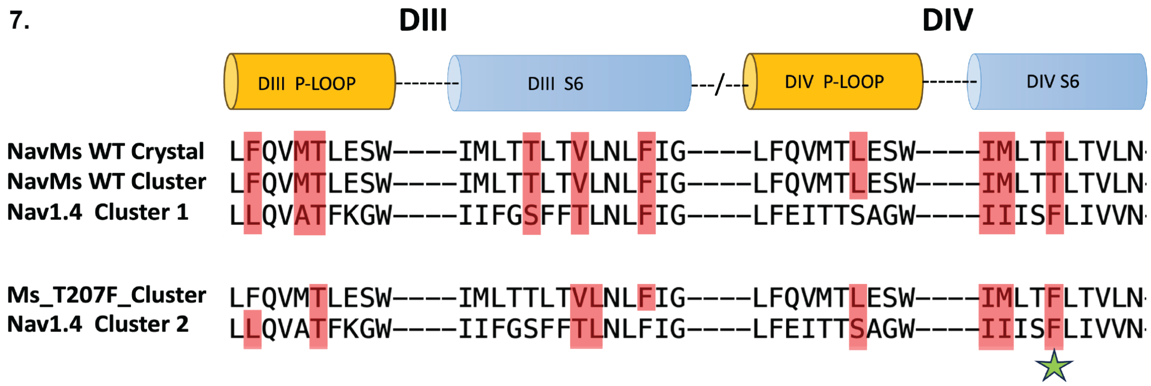

Supplementary Figure 7 Sequence alignment showing similarity of riluzole binding sites between NavMs and human Nav1.4 cluster 1, and NavMs (T207F) and Nav1.4 cluster 2 from the Nav1.4 DIII-DIV MD simulation

NavMs and Nav1.4 sequences aligned to the P-loop and S6 helix regions of Nav1.4 DIII and DIV that are involved in riluzole binding. Highlighted residues (red shade) represent amino acid residues in the DIII-DIV fenestration of Nav1.4, and their NavMs equivalents, that are ≤4Å from riluzole in the crystal structure or MD simulations. The green star represents the position of the LA binding site residue critical for riluzole suppression of I_{NaL}.

8.

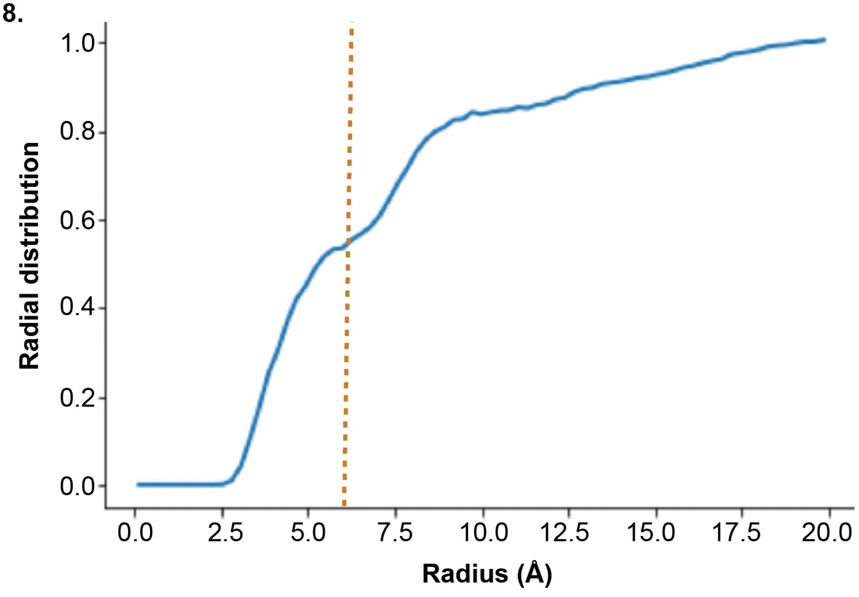

Supplementary Figure 8 Radial distribution function showing why the cutoff distance of 6A was used in MD

Supplementary Table 1: Crystallographic Data and Refinement Statistics

Data collection	**$NavMs_L$ (PDB ID 8S6J)**
Wavelength (Å)	2.7552
Space group	I422
Unit-cell parameters	
a, b, c (Å)	109.17,109.17,208.93
α, β, γ (°)	90, 90, 90
Resolution range (Å)	96.76 – 2.15 (2.21-2.15)
Total number of observations	557168 (36106)
Total number unique	34739 (2799))
Completeness	100.0 (100.0)
Multiplicity	16.0 (12.9)
<I/σ(I)>	18.2 (3.3)
CC(1/2)	0.979 (0.972)
$R_{merge\ all}$	0.072 (0.58)
$R_{pim\ All}$	0.017 (0.140)
Solvent content (%)	77.3
Molecule per ASU	1
Wilson B factor (Å^2)	43.9
Refinement	
Resolution Range (Å)	96.76 – 2.15
R_{work}	0.229
R_{free}	0.245
Reflection, working	34773
Reflection, free	1683
Average B factor (all atoms)	67.0
RMS bond angle	2.22
RMS bond length (Å)	0.0115
Ramachandran Analysis:	
Preferred region (%)	94.0
Allowed region (%)	6.0
Outliers (%)	0

Parentheses indicate statistics for the high-resolution data bin for x-ray data.

Supplementary Table 2: Activation and Inactivation of NavMS channels

		SSI $V_{1/2}$ (mV)	s	n	GV $V_{1/2}$ (mV)	s	n
**NavMs**	Vehicle	-84.5 ± 1.4	-3.3 ± 0.2	11	-40.9 ± 2.7	3.1 ± 0.2	5
	1 μM	-86.9 ± 2.6	-2.6 ± 0.1	5	-43.7 ± 3.4	3.3 ± 0.04	3
	2.5 μM	-94.1 ± 2.5*	-2.9 ± 0.1	18	-40.1 ± 1.5	3.5 ± 0.3	6
	5 μM	-95.5 ± 1.8*	-2.4 ± 0.09	8	-42.3 ± 4.0	2.9 ± 0.2	5
	25 μM	-97.3 ± 3.2*	-2.7 ± 0.2	6	-36.1 ± 1.8	3.1 ± 0.3	6
**NavMs T207A**	Vehicle	-84.7 ± 2.6	-3.4 ± 0.2	5	-37.8 ± 2.1	3.3 ± 0.3	5
	2.5 μM	-88.6 ± 1.6	-3.1 ± 0.2	6	-38.1 ± 1.6	3.3 ± 0.2	5

Average values for $V_{1/2}$ of activation (GV) and inactivation (SSI) ± S.E.M. as well as slope (s) from the Boltzmann function. * indicates $p < 0.05$ compared to vehicle, calculated by one-way ANOVA followed by post-hoc Tukey test. For SSI $V_{1/2}$ p = 0.025, 0.038 and 0.024 for 2.5, 5 and 25 μM riluzole respectively compared to vehicle.

Supplementary Table 3: Recovery from Inactivation for NavMS channels

		Tau Fast (s)	Tau Slow (s)	Fraction Tau fast	n
**NavMs**	Vehicle	0.009 ± 0.002	0.051 ± 0.006	0.56 ± 0.03	8
	2.5 μM	0.034 ± 0.007*	0.095 ± 0.026	0.50 ± 0.09	8
	5 μM	0.050 ± 0.004*	0.21 ± 0.09*	0.72 ± 0.09	5
	25 μM	0.033 ± 0.008*	0.088 ± 0.018	0.40 ± 0.08	5
**NavMs T207A**	Vehicle	0.011 ± 0.001	0.12 ± 0.04	0.68 ± 0.05	5
	2.5 μM	0.029 ± 0.006	0.086 ± 0.016	0.58 ± 0.11	5

Average values for τ_{fast} and τ_{slow} time constants ± S.E.M. calculated from double exponential fit of recovery data. * indicates $p < 0.05$ compared to vehicle, calculated by one-way ANOVA followed by post-hoc Tukey test. For τ_{fast} p = 0.008, 0.0002, and 0.027 for 2.5, 5 and 25 μM riluzole respectively, compared to vehicle. For τ_{slow} p = 0.013 for 5 μM riluzole compared to vehicle.

Supplementary Table 4: Primers used for mutagenesis of NavMs in this study

Name	Sequence
C52A_Forward	5'- GTGTGGATCAACTTGCTCTGACTATCTTTATTG -3'
C52A_Reverse	5'- CAATAAAGATAGTCAGAGCAAGTTGATCCACAC -3'
M204C_Forward	5'- CATCCCGTTCATCTGCCTCACCACCTTTAC –3'
M204C_Reverse	5'- GTAAAGGTGGTGAGGCAGATGAACGGGATG -3'
T207A_Forward	5'- CATCATGCTCACCGCCCTGACCGTG -3'
T207A_Reverse	5'- CACGGTCAGGGCGGTGAGCATGATG -3'
