# Supplementary material for: Structural basis for the rescue of hyperexcitable cells by the amyotrophic lateral sclerosis drug Riluzole
Source: Nat Commun. 2024 Sep 28;15:8426. doi: 10.1038/s41467-024-52539-4 (PMC11438954; doi:10.1038/s41467-024-52539-4)
Supplement: Supplementary file 3 — Legends for Supplementary Movies 1-4 [file 41467_2024_52539_MOESM3_ESM.docx]

**Legends for Supplementary Movies 1-4**

**Supplementary Movie 1. Riluzole interaction with WT NavMs.** Typical example of a riluzole molecule (coloured by heteroatom) trajectory from the WT MD simulation with riluzole binding in the fenestration of NavMs WT (pink) embedded in surrounding lipid (yellow). T207 residue highlighted with sidechain.

**Supplementary Movie 2. Riluzole interaction with NavMs T207A.** Typical example of a riluzole molecule (coloured by heteroatom) trajectory from the T207A MD simulation passing through the fenestration of NavMs T207A (blue) and into the pore. The T207A mutation is highlighted with sidechain and lipids are shown (yellow).

**Supplementary Movie 3. Riluzole interaction with NavMs containing T207F on one S6-helix element.** Riluzole molecule (coloured by heteroatom) trajectory from the T207F MD simulation where riluzole enters NavMs T207F (purple) through a WT fenestration (containing T207) then crosses the pore to bind stably in the T207F containing fenestration. NavMs residues at the S6 207 sites are highlighted by sidechain and lipids are shown (yellow).

**Supplementary Movie 4. Pathway taken by a typical riluzole from aqueous phase to fenestration.** Riluzole (coloured by heteroatom) passes into the membrane from the aqueous phase, sampling NavMs (grey) before accessing the fenestration opening (pink residues) to bind in the fenestration. Lipid heads (yellow) delineate the position of the membrane.
